# Antispasmodic effects of *Citrus aurantium* flowers aqueous extract on uterus of non-pregnant rats

**Published:** 2011

**Authors:** Akram Ahangarpour, Ali Akbar Oroojan, Ashraf Amirzargar, Maryam Ghanavati

**Affiliations:** 1 Department of Physiology, School of Medicine, Physiology and Diabetes Research Center, Ahvaz Jundishapur University of Medical Sciences, Ahvaz, Iran.; 2 Department of Physiology, School of Medicine, Ahvaz Jundishapur University of Medical Sciences, Ahvaz, Iran.; 3 Department of Nursing, School of Nursing and Midwifery, Ahvaz Jundishapur University of Medical Sciences, Ahvaz, Iran.

**Keywords:** *Citrus aurantium*, *Propranolol*, *Naloxone*, *Antispasmodic*, *Uterus*, *Rat*

## Abstract

**Background:**

Citrus aurantium is a small citrus tree, with scented white flowers. The *C. aurantium* is used in Asian herbal medicine primarily to treat digestive problems.

**Objective:**

The goal of this study is to investigate the effect of *C. aurantium* flower's aqueous extract on uterine contraction in presence of some known uterus stimulants.

**Materials and Methods:**

In experimental study 30 virgin Wistar rats 200-300gr were obtained. After laparatomy, a piece of Uterus was dissected out and mounted in an organ bath (10ml) containing De Jalon (29°C) and contracted by KCl (60mM), oxytocin (10mU/ml) and barium chloride (4mM) then the effect of *C. aurantium* flower's aqueous extract (1-8 mg/ml) on the uterine contractions was investigated.μμthe role of β-adrenoceptors, opioid receptors were evaluated.

**Results:**

Cumulative concentrations of the extract (1-8 mg/ml) decreased KCl, oxytocin and barium chloride induced uterine contractions, dose-dependently (p<0.001). *C. aurantium* flower's aqueous extract was unaffected on incubation the tissue with propranolol and naloxone.

**Conclusion:**

It seems that the extract induced antispasmodic effect mainly via calcium influx blockade. However, neither β-adrenoceptors nor opioid receptors were involved. Since the extract has antispasmodic effect on uterus contraction therefore we can suggest that more study will be necessary to relief dysmenorrheal.

## Introduction

*Citrus aurantium*, Seville or sour orange is a small citrus tree, with aroma white flowers. The most common use of *C. aurantium* is medicinal rather than culinary. The entire Raw fruit, is used in Asian herbal medicine primarily to Remedy digestive problems. There is thus a history of benign human consumption of *C. aurantium* fruit (1). This herb use for treatment of obesity and suppress appetite (2).* C. aurantium* has pharmacological applications that its flowers have been used as a sedation and heart tonic in traditional medicine (3). In humans, rats, guinea pigs, cats, and/or dogs, extracts of *C. aurantium* have been evaluated for effects on the cardiovascular system that include changes in blood pressure, cardiovascular toxicity, contractility and excitability of the heart muscle, and/or adrenergic activity (4). *C. aurantium* extract can increase the L-type calcium current in guinea pig ventricular myocytes significantly in a concentration-dependent manner, and promote the opening of calcium channel (5). Studies of the flowers of this natural medicine have reported that some constituents exhibited antioxidant activity by flavonoids containing and the aqueous extract showed inotropic effect. The *C. aurantium* extract had an effect in the hypertention (1, 2). The new products that called “ephedra free” usually contain C. aurantium extracts and it has 3 to 6% of p-synephrine. p-synephrine acts such as cardiac debit increase, peripheric vasoconstriction, broncodilatation and CNS stimulation (6). Since in traditional medicine the use of this plant was recommended in pregnancy therefore the present study was an attempt to investigate the effect of *C. aurantium* flower aqueous extract on uterine contraction and its possible mechanism.

## Materials and methods

**Plant extraction**

*C. aurantium* flower were purchased from local herbal shop in 2009 and were powdered by miller machine. Aqueous extract was accommodated by sokcele method using 30gr powder and 300ml distilled water for 8 hours. The complex was filtered with Whatman No1 filter paper. After that solution dried at 25°C to obtain powder and it was stored at Refrigerator until being used.

**Animals and tissue preparation**

30 Adult female Wistar rats (200-300gr) were obtained from Ahwaz Jundishapur University of Medical Sciences animal house and kept at 12- h light/ dark cycle and at 20-24°C were allowed free access to tap water and commercial chow. They were divided in to 5 groups (KCl-induced, barium chloride- induced, propranolol incubation, naloxane incubation and oxytocin-induced). 

In this study the rats were sacrificed by a sharp blow on the neck. After laparotomy uterus was removed and washed with cold and oxygenated with De Jalon solution and cut into 1.5cm long and then mounted in an organ bath containing De Jalon solution (10ml) between two stainless steel hooks vertically. One of the hooks was upper hook that connected to an isometric transducer (UF1 Harvard transducer, UK) and this transducer was connected to a recorder machine (Harvard Universal Oscillograph, UK) and another one was lower hook that fixed at the bottom of the organ bath. The De Jalon solution was contained NaCl (154), KCl (5.6), CaCl_2_ (0.3), NaHCO_3_ (1.7), MgCl_2_ (1.4) and glucose (5.55) (in mM) with pH of 7.4 and temperature of 29°C and the bubble of air was present in the De Jalon continuously (6). The piece of uterus was then Keep under 1g initial tension and Permit to Parity for 1h during which bath solution was washed every 15 min.

**Drugs**

Potassium chloride and barium chloride were purchased from Merck (Germany), naloxone (Tolid Daru Company, Tehran-Iran), propranolol (Tolid Daru Company, Tehran-Iran) and oxytocin (Weimer Pharma, Germany). 

*C. aurantium* extract powder and all chemicals were dissolved in De Jalon and added to organ bath, degree of drugs solution was equivalent with laboratories temperature (29ºC).

**Experimental protocols**

After equilibrium period, the uterus was contracted by KCL 60mM (7, 8) and barium chloride 4mM (9) in the plateau of contraction the concentrations of extract (1, 2, 4 and 8 mg/ml) were added cumulatively. For the study uterus opioid receptors, naloxone (1μM) and for the study of β-adrenoceptors receptors, propranolol (1μM) (10) were added to organ bath about 30 min without any refreshing for blocking these receptors in uterus tissue and after that concentrations of extract (1, 2, 4 and 8 mg/ml) were added cumulatively. Uterus tissue contracted by oxytocin (10 mU/ml) (10) and when oxytocin doesn't have plateau the tissue was washed. After that extract was added to organ bath without any dose of oxytocin and then percent's differences between peak amplitude of oxytocin was calculated. These protocols repeated for 6 rats in each groups. 

**Statistical analysis**

The mean±SEM of contraction forces induced by KCl, barium chloride and OT were regarded as (0%) and calculated for each group. The results were statistically analyzed by one way ANOVA and post hoc LSD tests and (p-values <0.05) was significant. The (n) was the number of animals that used in each group.

## Results

**Effect of cumulatively concentration of** ***C. aurantium* flowers aqueous extract after making uterus contractions with** **KCL (60mM) **

**Figure 1:**
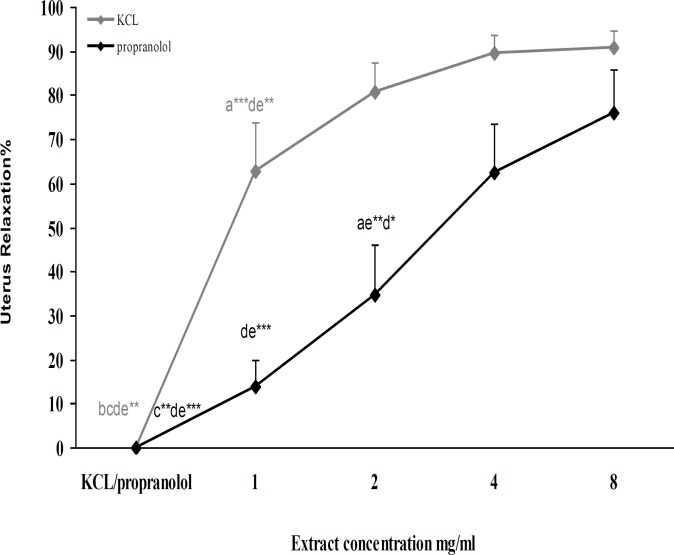
Effect of cumulative concentration of *C. aurantium* aqueous extract on KCL (60 mM) induced uterus contractions and after incubation with propranolol (1 μM) (n=6, **= p<0.01, ***= p<0.001).

KCl (60mM) induced made contraction in uterus tissue, after 2min when the contractions of uterus arrived to the plateau, the concentrations of C. aurantium flowers aqueous extract (1, 2, 4 and 8 mg/ml) was added cumulatively to the organ bath with 10 min intervals and without refreshing bath solution. KCl (60mM) had significantly difference with all of the concentrations of extracts (p<0.001) also the relaxation effect of 1mg/ ml extract was significantly different with 4 and 8 mg/ml (p<0.01) of extract on contraction that made by KCL (Figure 1).

**Effect of** ***C. aurantium*** **flowers aqueous extract activity after tissue incubation with propranolol**

After induced uterus contraction by KCl (60mM) and without refreshing bath, propranolol (1μM) added to organ bath for 30 min without any refreshing for blocking β-adrenoceptors, then concentrations of extract (1, 2, 4 and 8 mg/ml) were added cumulatively to the organ bath with 10 min intervals and without refreshing. 

There were significantly differences among propranolol (1μM) with 2 (p<0.01), 4 and 8 mg/ml (p<0.001) of extract. Relaxation effect of 1mg/ml extract had significant differences with 4 and 8 mg/ml (p<0.001) also this effect of 2mg/ml extract had significant differences with 4 (p<0.05) and 8 mg/ml (p<0.01) (Figure 1). 

**Effect of** ***C. aurantium*** **flowers aqueous extract on barium chloride-induced contraction**

**Figure 2:**
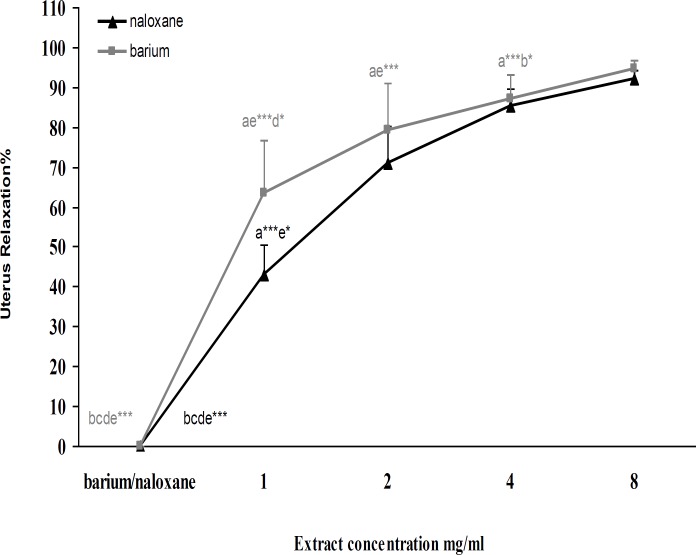
Effect of cumulative concentration of *C.* *aurantium* aqueous extract on barium chloride (4 mM) induced uterus contractions and after incubation with naloxone (1 μM) (n=6, *= p<0.05, ***= p<0.001).

After barium chloride (4mM) induced uterus contraction, C. aurantium flowers aqueous extract (1, 2, 4 and 8 mg/ml) was added cumulatively. Contractions barium chloride (4mM) had significant difference with another groups (p<0.001). Relaxation effect of 1 and 2 mg/ ml extract has significant difference with 8 mg/ml of extract (p<0.001). Also 1mg/ml of C. aurantium flowers aqueous extract had significant difference (p<0.05) with 4 mg/ml of extract (Figure 2).

**Effect of** ***C. aurantium*** **flowers aqueous extract activity after tissue incubation with naloxone**

 KCl (60mM) induced made contraction in uterus tissue after 2min interval, naloxone (1μM) as a non- selective opioid receptor antagonist added to organ bath for 30 min without refreshing. Then 1, 2, 4 and 8 mg/ml of extract were added to organ bath cumulatively and the interval was 10min. The result showed a significant difference between naloxone (1μM) and all groups (p<0.001) and relaxation effect of 1 mg/ml extract had significant differences with 8 mg/ml (p<0.05) group (Figure 2).

**Effect of** ***C. aurantium*** **flowers aqueous extract activity on oxytocin-induced uterus contraction**

**Figure 3:**
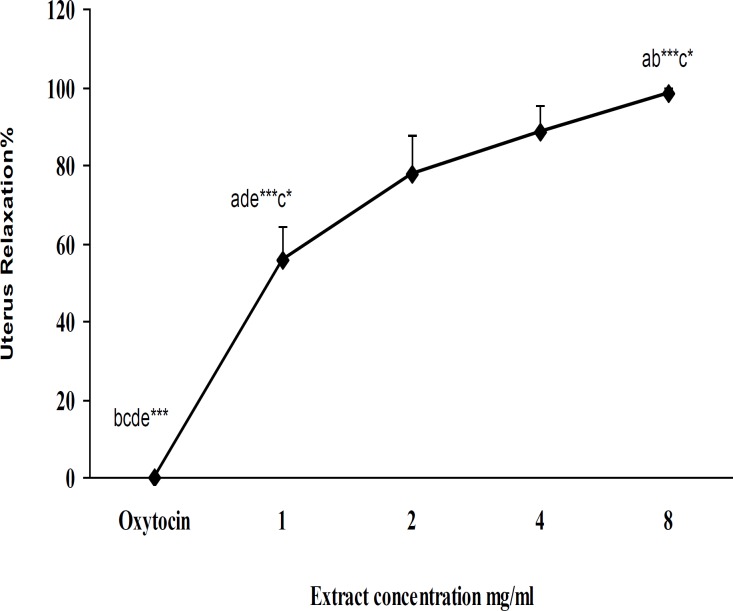
Effect of non-cumulative concentration of *C.* *aurantium* aqueous extract on rat uterus contracted by oxytocin (10mU/ ml)

First oxytocin (10mU/ml) was added to the bath with 3min interval, then uterus tissue in the bath was washed with De Jalon. Then 1mg/ml concentration of *C. aurantium* flowers aqueous extract was added to the bath for 3 min and without any refreshing, oxytocin was used with more than 3min interval. This protocol has been used for 2, 4 and 8 mg/ml concentration of extract. The time was about 15 min. After that all of the uterus tissue was washed. All of the concentrations of extract had shown relaxation effect with oxytocin (10mU/ml) group significantly (p<0.001). Also 1mg/ml of extract had significant difference with 2 (p<0.05), 4 and 8 mg/ml (p<0.001) and the difference between 2 mg/ml with 8 mg/ml (p<0.05) was significant (Figure 3).

a= difference between KCL group with other groups.

b= difference between 1mg/ml group with other groups.

c= difference between 2mg/ml group with other groups.

d= difference between 4mg/ml group with other groups.

e= difference between 8mg/ml group with other groups.

a= difference between barium chloride group with other groups.

b= difference between 1mg/ ml group with other groups.

c= difference between 2mg/ ml group with other groups.

d= difference between 4mg/ ml group with other groups.

e= difference between 8mg/ ml group with other groups.

(n=6, *= p<0.05, ***= p<0.001).

a= difference between oxytocin group with other groups.

b= difference between 1mg/ ml group with other groups.

c= difference between 2mg/ ml group with other groups.

d= difference between 4mg/ ml group with other groups.

e= difference between 8mg/ ml group with other groups.

## Discussion

Present results of this study showed that aqueous extract of *C.* *aurantium* flowers induces antispasmodic effect on the uterine muscle contraction caused by KCl, barium chloride or oxytocin. Also highest dose of the aqueous extract had most anticontractile effect on the uterus contraction. 

Propranolol is β-adrenoceptors antagonism and in some studies it seems clear that activation of β-adrenoceptors in uterine smooth muscle results in inhibition of myometrial contraction (11), after incubation with propranolol (1μM), antispasmodic effect of *C.* *aurantium* flowers aqueous extract decreased the contractile tension of uterine smooth muscle that made by KCl 60 mM and propranolol (1μM) incubated in nonpregnant virgin's rat. According to these results we can suggest that *C.* *aurantium* flowers aqueous extract doesn’t have any effect from β-adrenoceptors. in endometrial and myometrial regions of the uterus have been observed Opioid system expression (12). 

Opioid receptors activation relaxes uterus (13). Naloxone is a nonselective opioid receptors antagonist. In these results *C.* *aurantium* flowers aqueous extract activity had relaxant effect on uterus after incubation by naloxone (1μM). Therefore we can suggest that extract activity was not mediated via these receptors. Uterine smooth muscle contraction is mediated mainly via increased intracellular Ca^2+^ and is accomplished by excitation-contraction coupling mechanisms. A high K^+^ medium could depolarize the cellular membrane of uterine smooth muscle (14). 

Moreover, it is well-known that K+ induced contraction in smooth muscles is due to an increase in Ca^2+^ influx through voltage-operated Ca^2+^ channels. It has been suggested that any substance that inhibits high K^+^ induced contractions of smooth muscles is a blocker of Ca^2+^ influx (15). KCl induced uterine smooth muscle contraction results mainly from calcium influx via voltage-sensitive calcium channels (16). 

The present findings suggest that aqueous extract of *C.* *aurantium* flowers inhibits KCl induced uterine contractions acting on voltage-sensitive calcium channels, because this contraction is dependent on extracellular calcium. Oxytocin (OT) induces its effects via OT receptors (OTRs), which are members of the heptahelical family of G protein- coupled receptors. It is now well established that OT binding to its myometrial receptors leads to an increase in intracellular free calcium via the generation of inositol trisphosphate (17). 

The oxytocin stimulated increase in [Ca^2+^]_i_ is rapid and declines rather quickly in comparison to that seen in some other cell types, presumably as a result of active pumping of calcium out of the cell and back into the endoplasmic reticulum by the calcium transport ATPases (18). In this study, the effect of aqueous extract of *C.* *aurantium* flowers on contraction of uterus that has been made by oxytocin- induced was investigated. This investigation had done in a De Jalon solution and this solution had calcium concentration in itself. 

This extract reduce spontaneous motility and superimposing contractions with oxytocin- induced and this relaxation effect was dose- dependently that 8mg/ml of this extract had most relaxation effect in uterus muscle. Oxytocin also activates the L-type VDCCs (voltage dependent calcium channels) (17, 18). 

In our results, we demonstrated that aqueous extract of *C.* *aurantium* flowers reduced the oxytocin-induced contractions in De Jalon solution with calcium. Therefore we can suggest that the effect of this extract is via blockade L-type VDCCs that redound to relax the contracted of uterus tissue. 

Also we can say that voltage sensitive calcium channels have specific role in these contractions that decreased by using the concentrations of this extract. The effect of Barium chloride is inducing contraction in smooth muscle via blocking the potassium channels (19). Also, it has been reported that Ba^2+^ increases Ca^2+^ release from intracellular pools in smooth muscles and uterus (9). Our result showed that *C.* *aurantium* flowers extract reduced the contractions that induced by barium chloride dose- dependently, and this result was from prevention of releasing calcium or calcium efflux. 

In conclusion our result indicated that *C.* *aurantium* flowers aqueous extract induces antispasmodic effect on isolated rat uterus and this extract decreased the uterus contractions without involving β- adrenoceptors, and opioid receptors. These results indicate that *C.* *aurantium* flowers aqueous extract induces antispasmolytic effect on rat uterus mainly through blockage of the VDCCs. 

The precise mechanism of extract activity can be the study of Ca^2+^ alterations in the presence of the extract in animals or human myometrium. The antispasmodic effect of the plant’s extract observed in the present study, support the clinical efficacy and use of *C.* *aurantium* flowers in the treatment of dysmenorrhoea and other uterine spasmodic disorders. 

This process appears to be the most relevant physiologically and should be concentrated on in the future research.
